# Supplementary material for: Maternal hypertensive disorder of pregnancy and offspring early-onset cardiovascular disease in childhood, adolescence, and young adulthood: A national population-based cohort study
Source: PLoS Med. 2021 Sep 28;18(9):e1003805. doi: 10.1371/journal.pmed.1003805 (PMC8478255; doi:10.1371/journal.pmed.1003805)
Supplement: S1 Checklist — (DOCX) [file pmed.1003805.s001.docx]

**S2_STROBE Statement—Checklist of items that should be included in reports of cohort studies.**

|  | | Item No | Recommendation | | Page No |
| --- | --- | --- | --- | --- | --- |
| Title and abstract | | 1 | (*a*) Indicate the study’s design with a commonly used term in the title or the abstract | | Title and abstract |
|  |  |  | (*b*) Provide in the abstract an informative and balanced summary of what was done and what was found | |  |
| Introduction | | | | | |
| Background/rationale | | 2 | Explain the scientific background and rationale for the investigation being reported | | Introduction section: paragraph 1-2 |
| Objectives | | 3 | State specific objectives, including any prespecified hypotheses | | Introduction section: paragraph 3 |
| Methods | | | | | |
| Study design | | 4 | Present key elements of study design early in the paper | | Method section: subsection “Study design and participants” |
| Setting | | 5 | Describe the setting, locations, and relevant dates, including periods of recruitment, exposure, follow-up, and data collection | | Method section: subsection “Study design and participants” and “Maternal hypertensive disorders of pregnancy” |
| Participants | | 6 | (*a*) Give the eligibility criteria, and the sources and methods of selection of participants. Describe methods of follow-up | | Method section: subsection “Study design and participants”, Figure 1 |
|  |  |  | (*b*) For matched studies, give matching criteria and number of exposed and unexposed | |  |
| Variables | | 7 | Clearly define all outcomes, exposures, predictors, potential confounders, and effect modifiers. Give diagnostic criteria, if applicable | | Method section: subsection “Study design and participants”, “Maternal hypertensive disorders of pregnancy”, “Outcome of interest”, and “Covariates”, Appendix 4 |
| Data sources/ measurement | | 8* | For each variable of interest, give sources of data and details of methods of assessment (measurement). Describe comparability of assessment methods if there is more than one group | | Method section: subsection “Study design and participants”, “Maternal hypertensive disorders of pregnancy”, “Outcome of interest”, and “Covariates”, Appendix 4 |
| Bias | | 9 | Describe any efforts to address potential sources of bias | | Method section: subsection “Statistical analysis” |
| Study size | | 10 | Explain how the study size was arrived at | | Method section: subsection “Study design and participants” |
| Quantitative variables | | 11 | Explain how quantitative variables were handled in the analyses. If applicable, describe which groupings were chosen and why | | Method section: subsection “Covariates”, Appendix 4 |
| Statistical methods | | 12 | (*a*) Describe all statistical methods, including those used to control for confounding | | Method section: subsection “Covariates”, and “Statistical analysis” |
|  |  |  | (*b*) Describe any methods used to examine subgroups and interactions | |  |
|  |  |  | (*c*) Explain how missing data were addressed | |  |
|  |  |  | (*d*) If applicable, explain how loss to follow-up was addressed | |  |
|  |  |  | (*e*) Describe any sensitivity analyses | |  |
| Results | | | | |  |
| Participants | | 13* | (a) Report numbers of individuals at each stage of study—eg numbers potentially eligible, examined for eligibility, confirmed eligible, included in the study, completing follow-up, and analysed | | Results section: paragraph 1, Figure 1 |
|  |  |  | (b) Give reasons for non-participation at each stage | |  |
|  |  |  | (c) Consider use of a flow diagram | |  |
| Descriptive data | | 14* | (a) Give characteristics of study participants (eg demographic, clinical, social) and information on exposures and potential confounders | | Results section: paragraph 1-2, Table 1 |
|  |  |  | (b) Indicate number of participants with missing data for each variable of interest | |  |
|  |  |  | (c) Summarise follow-up time (eg, average and total amount) | |  |
| Outcome data | | 15* | Report numbers of outcome events or summary measures over time | | Results section: paragraph 2 |
| Main results | 16 | | | (*a*) Give unadjusted estimates and, if applicable, confounder-adjusted estimates and their precision (eg, 95% confidence interval). Make clear which confounders were adjusted for and why they were included | Results section: paragraph 2-4, Table 2-4, Figure 2 |
|  |  |  |  | (*b*) Report category boundaries when continuous variables were categorized |  |
|  |  |  |  | (*c*) If relevant, consider translating estimates of relative risk into absolute risk for a meaningful time period |  |
| Other analyses | 17 | | | Report other analyses done—eg. analyses of subgroups and interactions, and sensitivity analyses | Results section: paragraph 5, Table S3-S6, Figure S2-S3 |
| Discussion | | | | | |
| Key results | 18 | | | Summarise key results with reference to study objectives | Discussion section: paragraph 1 |
| Limitations | 19 | | | Discuss limitations of the study, taking into account sources of potential bias or imprecision. Discuss both direction and magnitude of any potential bias | Discussion section: paragraph 7 |
| Interpretation | 20 | | | Give a cautious overall interpretation of results considering objectives, limitations, multiplicity of analyses, results from similar studies, and other relevant evidence | Discussion section: paragraph 2-7 |
| Generalisability | 21 | | | Discuss the generalisability (external validity) of the study results | Discussion section: paragraph 7-8 |
| Other information | | | | | |
| Funding | 22 | | | Give the source of funding and the role of the funders for the present study and, if applicable, for the original study on which the present article is based | Funding section |

*Give information separately for exposed and unexposed groups.
