# Supplementary material for: Maternal hypertensive disorder of pregnancy and offspring early-onset cardiovascular disease in childhood, adolescence, and young adulthood: A national population-based cohort study
Source: PLoS Med. 2021 Sep 28;18(9):e1003805. doi: 10.1371/journal.pmed.1003805 (PMC8478255; doi:10.1371/journal.pmed.1003805)
Supplement: S1 Text — (DOCX) [file pmed.1003805.s002.docx]

**S1_Study_protocol**

**Maternal hypertensive disorder of pregnancy and offspring early-onset cardiovascular disease in childhood, adolescence, and young adulthood: A national population-based cohort study**

1. **Background**

Cardiovascular diseases (CVDs) remain the leading cause of death worldwide [1,2]. Although substantial progress has been made in the prevention and treatment of CVD over the past decades, the rising prevalence of CVD has been observed in children and young adults [3-9]. Previous studies have identified a wide range of risk factors for cardiovascular disease, such as smoking, obesity and stress [10,11]. Developing bodies of evidence has emerged concerning non-genetic intergenerational connections between maternal illness during pregnancy and risk factors for CVD among offspring [12-14].

Hypertensive disorders of pregnancy (HDP) is an important cause of morbidity and mortality in pregnant women and complicates about 10% of pregnancies globally [15]. Offspring of mothers with HDP have a higher prevalence of CVD risk factors, including higher blood pressure, obesity, abnormal distribution of lipid profile, and disorders of glucose metabolism [16,17]. We aimed to examine the associations between maternal HDP and early-onset CVD in offspring, and whether co-existing maternal CVD and maternal diabetes further increased the risk of CVD among offspring.

1. **Data sources**

A unique civil personal identification number is assigned for all residents in Denmark, which permits individual-level linkage across various national registries [18].

1. **Study population and period**

We will include all live-born children in Denmark between 1977 and 2018, after excluding offspring with diagnosed congenital heart disease. The follow-up began at birth and ended until the first-time diagnosis of CVD, emigration, death, or 31 December 2018, whichever came first.

1. **Variable definitions**
   1. **Exposures**

Information on maternal HDP was retrieved from the Danish National Patient Register (DNPR), using the *International Classification of Disease* [ICD] (ICD-8, 1978–1993; ICD-10, 1994 forward). Hypertension in Pregnancy was classified as pre-gestational hypertension, gestational hypertension, pre-eclampsia and eclampsia, pre-eclampsia was further categorized into unspecified eclampsia, moderate eclampsia, severe eclampsia and HELLP (hemolysis, elevated liver enzymes and low platelet count) syndrome according to the severity.

- 1. **Outcomes**

4.2.1 Primary outcome: Overall CVD in offspring ((ICD-8 codes: 390-444.1, 444.3-458, 782.4; ICD-10 codes: I00-I99).

4.2.2 Secondary outcome: Type-specific CVD including myocardial infarction, cerebrovascular disease, stroke, heart failure, atrial fibrillation, hypertensive disease, deep vein thrombosis, pulmonary embolism, rheumatic heart disease, and peripheral arterial disease.

- 1. **Covariates**
- sex (male, female)
- singleton (yes, no)
- birth year of the child (1977-1980, 5-year intervals during 1981-2015, and 2016-2018)
- maternal age (<20, 20-24, 25-29, 30-34, or ≥35 years)
- maternal education (0-9, 10-14, or ≥15 years)
- maternal income at birth (no income, 3 tertiles)
- maternal pre-pregnancy BMI (underweight <18.5, normal 18.5-24.9, overweight 25.0-29.9, obese ≥30.0)
- maternal smoking during pregnancy (yes or no)
- parity (1, 2, or ≥3 children)
- maternal cohabitation (single or cohabitating)
- maternal residence (Copenhagen, cities with ≥100,000 inhabitants, or other)
- maternal history of diabetes before childbirth (yes or no)
- maternal history of CVD before childbirth (yes or no)
- parental history of CVD before childbirth (yes or no)

1. **Statistical analyses**

- Kaplan-Meier method was performed to estimate cumulative incidence in different groups.
- Cox regression models was used to estimate hazard ratios (HRs) and 95% CIs to assess the association between maternal HDP and overall/type-specific CVD in offspring. The proportional hazards assumption was assessed graphically using the log-minus-log plot.
- We assessed whether the association between maternal HDP and offspring CVD varied by the maternal history of CVD or diabetes through examining the interaction term between maternal HDP and maternal history of CVD, and the interaction term between maternal HDP and maternal history of diabetes.
- Assessed the association by timing of onset and severity of pre-eclampsia (moderate, severe eclampsia, and HELLP syndrome).
- Other sensitivity analysis:
  - Sibship analysis by restricting offspring to sibling pairs born to same mother but different father (half-sibling) or same father and mother (full-sibling).
  - Evaluated whether timing of delivery would affect the observed associations by dividing offspring to preterm birth and term birth.
  - Stratified analysis by baseline characteristics.
  - Association between paternal hypertension and offspring CVD.
  - Association according to the timing of diagnosis of maternal HDP since childbirth.

**References**

1. Global, regional, and national disability-adjusted life-years (DALYs) for 315 diseases and injuries and healthy life expectancy (HALE), 1990-2015: a systematic analysis for the Global Burden of Disease Study 2015. Lancet (London, England). 2016;388(10053):1603-58. Epub 2016/10/14. doi: 10.1016/s0140-6736(16)31460-x. PubMed PMID: 27733283; PubMed Central PMCID: PMCPMC5388857.

2. Thomas H, Diamond J, Vieco A, Chaudhuri S, Shinnar E, Cromer S, et al. Global Atlas of Cardiovascular Disease 2000-2016: The Path to Prevention and Control. Global heart. 2018;13(3):143-63. Epub 2018/10/12. doi: 10.1016/j.gheart.2018.09.511. PubMed PMID: 30301680.

3. Anand SS, Yusuf S. Stemming the global tsunami of cardiovascular disease. Lancet. 2011;377(9765):529-32. Epub 2011/02/08. doi: 10.1016/S0140-6736(10)62346-X. PubMed PMID: 21295845.

4. Danaei G, Singh GM, Paciorek CJ, Lin JK, Cowan MJ, Finucane MM, et al. The global cardiovascular risk transition: associations of four metabolic risk factors with national income, urbanization, and Western diet in 1980 and 2008. Circulation. 2013;127(14):1493-502, 502e1-8. Epub 2013/03/14. doi: 10.1161/CIRCULATIONAHA.113.001470. PubMed PMID: 23481623; PubMed Central PMCID: PMCPMC4181853.

5. Ford ES, Ajani UA, Croft JB, Critchley JA, Labarthe DR, Kottke TE, et al. Explaining the decrease in U.S. deaths from coronary disease, 1980-2000. The New England journal of medicine. 2007;356(23):2388-98. Epub 2007/06/08. doi: 10.1056/NEJMsa053935. PubMed PMID: 17554120.

6. Nabel EG, Braunwald E. A tale of coronary artery disease and myocardial infarction. N Engl J Med. 2012;366(1):54-63. Epub 2012/01/06. doi: 10.1056/NEJMra1112570. PubMed PMID: 22216842.

7. Schmidt M, Jacobsen JB, Lash TL, Botker HE, Sorensen HT. 25 year trends in first time hospitalisation for acute myocardial infarction, subsequent short and long term mortality, and the prognostic impact of sex and comorbidity: a Danish nationwide cohort study. BMJ (Clinical research ed). 2012;344:e356. Epub 2012/01/27. doi: 10.1136/bmj.e356. PubMed PMID: 22279115; PubMed Central PMCID: PMCPMC3266429.

8. George MG, Tong X, Kuklina EV, Labarthe DR. Trends in stroke hospitalizations and associated risk factors among children and young adults, 1995-2008. Ann Neurol. 2011;70(5):713-21. Epub 2011/09/08. doi: 10.1002/ana.22539. PubMed PMID: 21898534.

9. George MG, Tong X, Bowman BA. Prevalence of Cardiovascular Risk Factors and Strokes in Younger Adults. JAMA Neurol. 2017;74(6):695-703. Epub 2017/04/11. doi: 10.1001/jamaneurol.2017.0020. PubMed PMID: 28395017; PubMed Central PMCID: PMCPMC5559660.

10. Jousilahti P, Tuomilehto J, Vartiainen E, Pekkanen J, Puska P. Body weight, cardiovascular risk factors, and coronary mortality. 15-year follow-up of middle-aged men and women in eastern Finland. Circulation. 1996;93(7):1372-9. Epub 1996/04/01. PubMed PMID: 8641026.

11. Riaz H, Khan MS, Siddiqi TJ, Usman MS, Shah N, Goyal A, et al. Association Between Obesity and Cardiovascular Outcomes: A Systematic Review and Meta-analysis of Mendelian Randomization Studies. JAMA network open. 2018;1(7):e183788. Epub 2019/01/16. doi: 10.1001/jamanetworkopen.2018.3788. PubMed PMID: 30646365; PubMed Central PMCID: PMCPMC6324374.

12. Persson M, Razaz N, Edstedt Bonamy AK, Villamor E, Cnattingius S. Maternal Overweight and Obesity and Risk of Congenital Heart Defects. J Am Coll Cardiol. 2019;73(1):44-53. Epub 2019/01/10. doi: 10.1016/j.jacc.2018.10.050. PubMed PMID: 30621950.

13. Tyrrell J, Richmond RC, Palmer TM, Feenstra B, Rangarajan J, Metrustry S, et al. Genetic Evidence for Causal Relationships Between Maternal Obesity-Related Traits and Birth Weight. Jama. 2016;315(11):1129-40. Epub 2016/03/16. doi: 10.1001/jama.2016.1975. PubMed PMID: 26978208; PubMed Central PMCID: PMCPMC4811305.

14. Gaillard R. Maternal obesity during pregnancy and cardiovascular development and disease in the offspring. Eur J Epidemiol. 2015;30(11):1141-52. Epub 2015/09/18. doi: 10.1007/s10654-015-0085-7. PubMed PMID: 26377700; PubMed Central PMCID: PMCPMC4684830.

15. Kassebaum NJ, Bertozzi-Villa A, Coggeshall MS, Shackelford KA, Steiner C, Heuton KR, et al. Global, regional, and national levels and causes of maternal mortality during 1990-2013: a systematic analysis for the Global Burden of Disease Study 2013. Lancet (London, England). 2014;384(9947):980-1004. Epub 2014/05/07. doi: 10.1016/s0140-6736(14)60696-6. PubMed PMID: 24797575; PubMed Central PMCID: PMCPMC4255481.

16. Davis EF, Lewandowski AJ, Aye C, Williamson W, Boardman H, Huang RC, et al. Clinical cardiovascular risk during young adulthood in offspring of hypertensive pregnancies: insights from a 20-year prospective follow-up birth cohort. BMJ Open. 2015;5(6):e008136. Epub 2015/06/25. doi: 10.1136/bmjopen-2015-008136. PubMed PMID: 26105032; PubMed Central PMCID: PMCPMC4480003.

17. Davis EF, Lazdam M, Lewandowski AJ, Worton SA, Kelly B, Kenworthy Y, et al. Cardiovascular risk factors in children and young adults born to preeclamptic pregnancies: a systematic review. Pediatrics. 2012;129(6):e1552-61. Epub 2012/05/23. doi: 10.1542/peds.2011-3093. PubMed PMID: 22614768.

18. Sorensen HT, Christensen T, Schlosser HK, Pedersen L. Use of medical databases in clinical epidemiology. ed n, editor. Aarhus, Denmark: SUN-TRYK: Aarhus Universitet; 2009.
