# Supplementary material for: Maternal hypertensive disorder of pregnancy and offspring early-onset cardiovascular disease in childhood, adolescence, and young adulthood: A national population-based cohort study
Source: PLoS Med. 2021 Sep 28;18(9):e1003805. doi: 10.1371/journal.pmed.1003805 (PMC8478255; doi:10.1371/journal.pmed.1003805)
Supplement: S2 Text — (DOCX) [file pmed.1003805.s003.docx]

**S2 Text**. **Detailed description of registers used in this study.**

Denmark has been providing public welfare including universal health care, education, student assistance, disability pensions and unemployment insurance through tax-funded services. There are population-based health databases in Denmark that regularly collect high-quality data including individual medical data and all live births and new residents in Denmark are assigned a unique 10-digit individual personal identification number (Central Personal Register number, CPR) since the 1960s. Information across different national registers are linked by the CPR number.

The Danish Civil Registration System (CRS)

The Danish Civil Registration System (CRS) [1] has been established since 1968, and collects information on date of birth, emigration, and immigration, sex, and other vital status in a daily basis.

The Danish Registry of Causes of Death

The Danish Registry of Causes of Death [4] has been computerized since 1970 and the non-electronic data on deaths has been collected since1924. This system has been collecting date of death, and immediate cause, underlying cause and contributory causes of death by law in Denmark. The ICD-8 was used to classify the causes of death during 1970-1993 and ICD-10 from 1994.

The Danish National Patient Register (DNPR)

The Danish National Patient Register (DNPR) [3] was established in 1977 and collects data from inpatient, outpatient, and emergency hospital record (each visit for one record in register). Information includes date of contact, primary and secondary discharge diagnoses, date and procedures of surgery, certain treatment in hospitals, and other hospital related information. Diagnoses are classified according to the ICD-8 codes (*International Classification of Disease* codes, 8th revision) and ICD-10 codes thereafter.

The Danish Medical Birth Registry (MBR)

The Danish Medical Birth Registry (MBR) [2] includes CPR number of new births, mothers, and fathers, as well as information on date of birth, birth characteristics such as sex, birth weight, gestational age, maternal characteristics such as maternal pre-pregnancy body mass index, maternal smoking during pregnancy. MBR was established in 1968 and has been computerized since 1973.

The Danish Integrated Database for Longitudinal Labour Market Research

The Danish Integrated Database for Longitudinal Labour Market Research was established in 1981 and contains labour market and socioeconomic data.

**References**

1. Schmidt M, Pedersen L, Sørensen HT. The Danish Civil Registration System as a tool in epidemiology. Eur J Epidemiol. 2014;29(8):541-9. Epub 2014/06/27. doi: 10.1007/s10654-014-9930-3. PubMed PMID: 24965263.

2. Knudsen LB, Olsen J. The Danish Medical Birth Registry. Dan Med Bull. 1998;45(3):320-3. Epub 1998/07/24. PubMed PMID: 9675544.

3. Lynge E, Sandegaard JL, Rebolj M. The Danish National Patient Register. Scand J Public Health. 2011;39(7 Suppl):30-3. Epub 2011/08/04. doi: 10.1177/1403494811401482. PubMed PMID: 21775347.

4. Petersson F, Baadsgaard M, Thygesen LC. Danish registers on personal labour market affiliation. Scand J Public Health. 2011;39(7 Suppl):95-8. Epub 2011/08/04. doi: 10.1177/1403494811408483. PubMed PMID: 21775363.
