# Supplementary material for: Maternal hypertensive disorder of pregnancy and offspring early-onset cardiovascular disease in childhood, adolescence, and young adulthood: A national population-based cohort study
Source: PLoS Med. 2021 Sep 28;18(9):e1003805. doi: 10.1371/journal.pmed.1003805 (PMC8478255; doi:10.1371/journal.pmed.1003805)
Supplement: S3 Text — (DOCX) [file pmed.1003805.s004.docx]

**S3 Text**. **Detailed description of covariates.**

Information on maternal and birth characteristics was retrieved from the Danish Medical Birth Register [1-3], while socioeconomic factors were retrieved from the Danish Integrated Database for Longitudinal Labour Market Research and the Danish Civil Registry System [4]. Information on parental history of CVD was obtained from the Danish National Patient Registry [3]. These included sex (male, female), singleton (yes, no), birth year of the child (1977-1980, 5-year intervals during 1981-2015, and 2016-2018), maternal age (<20, 20-24, 25-29, 30-34, or ≥35 years), maternal education (0-9, 10-14, or ≥15 years), maternal income at birth (no income, 3 tertiles), maternal pre-pregnancy BMI (underweight <18.5, normal 18.5-24.9, overweight 25.0-29.9, obese ≥30.0), maternal smoking during pregnancy (yes or no), parity (1, 2, or ≥3 children), maternal cohabitation (single or cohabitating), maternal residence (Copenhagen, cities with ≥100,000 inhabitants, or other), and maternal history of diabetes, maternal and parental history of CVD before childbirth (yes or no). A missing indicator method was used to deal with missing values.

**References**

1. Schmidt M, Pedersen L, Sørensen HT. The Danish Civil Registration System as a tool in epidemiology. Eur J Epidemiol. 2014;29(8):541-9. Epub 2014/06/27. doi: 10.1007/s10654-014-9930-3. PubMed PMID: 24965263.

2. Knudsen LB, Olsen J. The Danish Medical Birth Registry. Dan Med Bull. 1998;45(3):320-3. Epub 1998/07/24. PubMed PMID: 9675544.

3. Lynge E, Sandegaard JL, Rebolj M. The Danish National Patient Register. Scand J Public Health. 2011;39(7 Suppl):30-3. Epub 2011/08/04. doi: 10.1177/1403494811401482. PubMed PMID: 21775347.

4. Petersson F, Baadsgaard M, Thygesen LC. Danish registers on personal labour market affiliation. Scand J Public Health. 2011;39(7 Suppl):95-8. Epub 2011/08/04. doi: 10.1177/1403494811408483. PubMed PMID: 21775363.
