# Supplementary material for: Maternal hypertensive disorder of pregnancy and offspring early-onset cardiovascular disease in childhood, adolescence, and young adulthood: A national population-based cohort study
Source: PLoS Med. 2021 Sep 28;18(9):e1003805. doi: 10.1371/journal.pmed.1003805 (PMC8478255; doi:10.1371/journal.pmed.1003805)
Supplement: S1 Table — (DOCX) [file pmed.1003805.s005.docx]

**S1 Table.** **Exposure classification of hypertensive disorders from the International Classification of Diseases, the 8th and 10th version (ICD-8 and ICD-10) ^a^**

|  | **ICD-8** | **ICD-10** |
| --- | --- | --- |
| Preeclampsia and eclampsia |  |  |
| Moderate preeclampsia | 63703 | O14.0 |
| Severe preeclampsia | 63704 | O14.1 |
| HELLP syndrome |  | O14.2 |
| Unspecified preeclampsia | 63709 | O14.9 |
| Eclampsia | 63719 | O15 |
| Hypertensive disorders  Gestational hypertension |  |  |
| Gestational (pregnancy-induced) hypertension | 63700 | O13 |
| Unspecified maternal hypertension | 76029 | O16 |
| Pre-gestational hypertension |  |  |
| Essential hypertension | 40009 | I10 |
| Hypertensive heart disease | 40019  40029 | I11 |
| Hypertensive renal disease | 40039 | I12 |
| Hypertensive heart and renal disease | 40099 | I13 |
| Secondary hypertension | 40199 | I15 |
| Pre-existing hypertension complicating pregnancy |  | O10 |
| Pre-eclampsia superimposed on chronic hypertension |  | O11 |

^a^ Information on maternal hypertensive disorders came from the Danish National Patient Register.
