# Supplementary material for: Maternal hypertensive disorder of pregnancy and offspring early-onset cardiovascular disease in childhood, adolescence, and young adulthood: A national population-based cohort study
Source: PLoS Med. 2021 Sep 28;18(9):e1003805. doi: 10.1371/journal.pmed.1003805 (PMC8478255; doi:10.1371/journal.pmed.1003805)
Supplement: S2 Table — (DOCX) [file pmed.1003805.s006.docx]

**S2 Table. Outcome classification of overall CVD and specific CVD from the International Classification of Diseases, the 8th and 10th version (ICD-8 and ICD-10) ^a^**

|  | **ICD-8** | **ICD-10** | **Procedure/Surgery Codes** |
| --- | --- | --- | --- |
| Overall cardiovascular disease (CVD) | 390-444.1, 444.3-458, 782.4 | I00-I99 | 30350, 30354, 30240, KFNG, KFNF 30009, 30019, 30029, 30039, 30049, 30059, 30069, 30079, 30089, 30099, 30109, 30119, 30120, 30129, 30139, 30149, 30159, 30169, 30179, 30189, 30199, 30200, KFNA-KFNE, KFNH20 |
| Myocardial infarction | 410 | I21 |  |
| Cerebrovascular disease | 430-438 | I60-I69 |  |
| Stroke | 430-436 | I61-I64 |  |
| Heart failure | 427.0,427.1,782.4 | I110, I130, I132, I50 |  |
| Atrial fibrillation | 427.93, 427.94 | I48 |  |
| Hypertensive disease | 400-404 | I10-I15 |  |
| Deep vein thrombosis | 451.00 | I80.1-I80.3 |  |
| Pulmonary embolism | 450.99 | I26 |  |
| Rheumatic heart disease | 391, 393-398 | I01, I05-09 |  |
| Peripheral arterial disease | 443.89-443.99 | I73.9 |  |

^a^ The information on cardiovascular disease is from the Danish National Patient Registry or the Danish Cause of Death Register**.**
