# Supplementary material for: Maternal hypertensive disorder of pregnancy and offspring early-onset cardiovascular disease in childhood, adolescence, and young adulthood: A national population-based cohort study
Source: PLoS Med. 2021 Sep 28;18(9):e1003805. doi: 10.1371/journal.pmed.1003805 (PMC8478255; doi:10.1371/journal.pmed.1003805)
Supplement: S5 Table — (DOCX) [file pmed.1003805.s009.docx]

**S5 Table. Associations between maternal hypertensive disorder of pregnancy and early-onset CVD in offspring, by characteristics**

| **Characteristics** | **Total Maternal HDP** | | | **Preeclampsia or Eclampsia** | | | **Hypertension** | | |
| --- | --- | --- | --- | --- | --- | --- | --- | --- | --- |
|  | **No. Of CVD cases** | **Rate (1/10^3^)** | **HR (95%CI)** ^a^ | **No. Of CVD cases** | **Rate (1/10^3^)** | **HR (95%CI)** | **No. Of CVD cases** | **Rate (1/10^3^)** | **HR (95%CI)** |
| **Singleton** | | | | | | | | | |
| No | 245 | 2.07 | 1.14 (1.00-1.30) | 206 | 2.08 | 1.13 (0.98-1.30) | 39 | 2.03 | 1.22 (0.89-1.67) |
| Yes | 4,287 | 2.50 | 1.23 (1.20-1.27) | 3,166 | 2.56 | 1.22 (1.18-1.27) | 1,121 | 2.33 | 1.26 (1.19-1.34) |
| **Gender** | | | | | | | | | |
| Boy | 2,242 | 2.35 | 1.25 (1.20-1.30) | 1,664 | 2.40 | 1.24 (1.18-1.3) | 578 | 2.23 | 1.29 (1.19-1.40) |
| Girl | 2,290 | 2.60 | 1.21 (1.16-1.26) | 1,708 | 2.67 | 1.2 (1.14-1.26) | 582 | 2.41 | 1.22 (1.13-1.33) |
| **Maternal parity** | | | | | | | | | |
| 1 | 2,621 | 2.40 | 1.2 (1.15-1.25) | 2,075 | 2.42 | 1.19 (1.14-1.24) | 546 | 2.30 | 1.24 (1.14-1.35) |
| 2 | 1,239 | 2.54 | 1.27 (1.20-1.35) | 848 | 2.65 | 1.27 (1.18-1.35) | 391 | 2.34 | 1.29 (1.17-1.43) |
| ≥3 | 672 | 2.63 | 1.25 (1.16-1.36) | 449 | 2.81 | 1.27 (1.15-1.39) | 223 | 2.34 | 1.23 (1.08-1.40) |
| **Maternal age at childbirth (years)** | | | | | | | | | |
| <20 | 182 | 2.99 | 1.07 (0.92-1.24) | 156 | 2.91 | 1.03 (0.88-1.21) | 26 | 3.56 | 1.30 (0.88-1.91) |
| 20-24 | 1,221 | 2.99 | 1.22 (1.15-1.29) | 985 | 2.95 | 1.20 (1.12-1.28) | 236 | 3.17 | 1.32 (1.16-1.50) |
| 25-29 | 1,578 | 2.44 | 1.24 (1.18-1.31) | 1,193 | 2.46 | 1.24 (1.17-1.31) | 385 | 2.38 | 1.27 (1.14-1.40) |
| 30-34 | 1,007 | 2.18 | 1.25 (1.18-1.33) | 694 | 2.26 | 1.26 (1.16-1.35) | 313 | 2.03 | 1.24 (1.11-1.39) |
| 35+ | 544 | 2.11 | 1.22 (1.12-1.33) | 344 | 2.21 | 1.23 (1.11-1.38) | 200 | 1.95 | 1.20 (1.04-1.38) |
| **Maternal smoking during pregnancy**^c^ | | | | | | | | | |
| No | 1,265 | 1.68 | 1.21 (1.15-1.29) | 886 | 1.75 | 1.24 (1.16-1.33) | 379 | 1.54 | 1.16 (1.04-1.28) |
| Yes | 268 | 1.75 | 1.09 (0.97-1.23) | 193 | 1.79 | 1.09 (0.95-1.26) | 75 | 1.66 | 1.09 (0.87-1.37) |
| Unknown | 2,999 | 3.22 | 1.25 (1.20-1.29) | 2,293 | 3.18 | 1.22 (1.17-1.27) | 706 | 3.38 | 1.34 (1.25-1.44) |
| **Maternal education at childbirth, years** | | | | | | | | | |
| 0-9 | 1,888 | 3.09 | 1.24 (1.18-1.29) | 1,454 | 3.08 | 1.21 (1.15-1.28) | 434 | 3.13 | 1.32 (1.20-1.45) |
| 10-14 | 1,779 | 2.21 | 1.20 (1.14-1.26) | 1,320 | 2.26 | 1.19 (1.13-1.26) | 459 | 2.07 | 1.21 (1.10-1.32) |
| 15+ | 831 | 2.04 | 1.28 (1.20-1.38) | 577 | 2.13 | 1.30 (1.20-1.42) | 254 | 1.87 | 1.24 (1.10-1.41) |
| Unknown | 34 | 2.47 | 1.14 (0.81-1.61) | 21 | 2.07 | 0.95 (0.62-1.47) | 13 | 3.59 | 1.68 (0.97-2.90) |
| **Maternal cohabitation at childbirth** | | | | | | | | | |
| No | 2,057 | 2.41 | 1.21 (1.15-1.26) | 1,577 | 2.46 | 1.2 (1.14-1.26) | 480 | 2.25 | 1.25 (1.14-1.36) |
| Yes | 2,475 | 2.52 | 1.25 (1.20-1.30) | 1,795 | 2.59 | 1.24 (1.18-1.3) | 680 | 2.37 | 1.27 (1.17-1.37) |
| **Maternal residence at childbirth** | | | | | | | | | |
| Copenhagen | 357 | 2.25 | 1.16 (1.05-1.29) | 287 | 2.39 | 1.19 (1.06-1.34) | 70 | 1.80 | 1.05 (0.83-1.33) |
| Big cities≥100 000 inhabitants | 544 | 2.30 | 1.30 (1.19-1.41) | 344 | 2.25 | 1.28 (1.15-1.42) | 200 | 2.39 | 1.33 (1.16-1.53) |
| Others | 3,631 | 2.52 | 1.22 (1.18-1.27) | 2,741 | 2.58 | 1.21 (1.17-1.26) | 890 | 2.36 | 1.26 (1.18-1.35) |
| **Maternal CVD history before childbirth** | | | | | | | | | |
| No | 4,397 | 2.46 | 1.23 (1.19-1.26) | 3,286 | 2.52 | 1.22 (1.18-1.26) | 1,111 | 2.30 | 1.25 (1.18-1.33) |
| Yes | 135 | 2.83 | 1.29 (1.09-1.54) | 86 | 2.81 | 1.21 (0.98-1.51) | 49 | 2.85 | 1.46 (1.10-1.94) |
| **Paternal CVD history before birth of the child** | | | | | | | | | |
| No | 4,375 | 2.47 | 1.23 (1.19-1.27) | 3,260 | 2.52 | 1.22 (1.18-1.26) | 1,115 | 2.31 | 1.26 (1.18-1.33) |
| Yes | 122 | 2.54 | 1.24 (1.03-1.49) | 84 | 2.54 | 1.21 (0.97-1.50) | 38 | 2.55 | 1.32 (0.96-1.82) |
| Unknown | 35 | 2.39 | 1.20 (0.85-1.68) | 28 | 2.48 | 1.22 (0.84-1.78) | 7 | 2.07 | 1.11 (0.53-2.33) |
| **Maternal DM history before childbirth** | | | | | | | | | |
| No | 4,376 | 2.46 | 1.23 (1.19-1.27) | 3,265 | 2.51 | 1.21 (1.17-1.26) | 1,111 | 2.33 | 1.26 (1.19-1.34) |
| Yes | 156 | 2.62 | 1.24 (1.05-1.47) | 107 | 2.89 | 1.31 (1.07-1.60) | 49 | 2.17 | 1.11 (0.83-1.48) |

Abbreviations: HDP, hypertensive disorders of pregnancy; CVD, cardiovascular disease; cHR, crude hazard ratio; aHR, adjusted hazard ratio.

^a^Adjusted for calendar year, sex, singleton status, parity, maternal age, maternal smoking, maternal education, maternal cohabitation, maternal country of origin, maternal income at birth, maternal BMI, maternal residence at birth, maternal history of CVD and diabetes before childbirth, and paternal history of CVD before childbirth.
