# Supplementary material for: Maternal hypertensive disorder of pregnancy and offspring early-onset cardiovascular disease in childhood, adolescence, and young adulthood: A national population-based cohort study
Source: PLoS Med. 2021 Sep 28;18(9):e1003805. doi: 10.1371/journal.pmed.1003805 (PMC8478255; doi:10.1371/journal.pmed.1003805)
Supplement: S1 Fig — (DOCX) [file pmed.1003805.s011.docx]

**S1 Fig. Causal diagram showing selection of covariates for confounding control. ^a^**

**
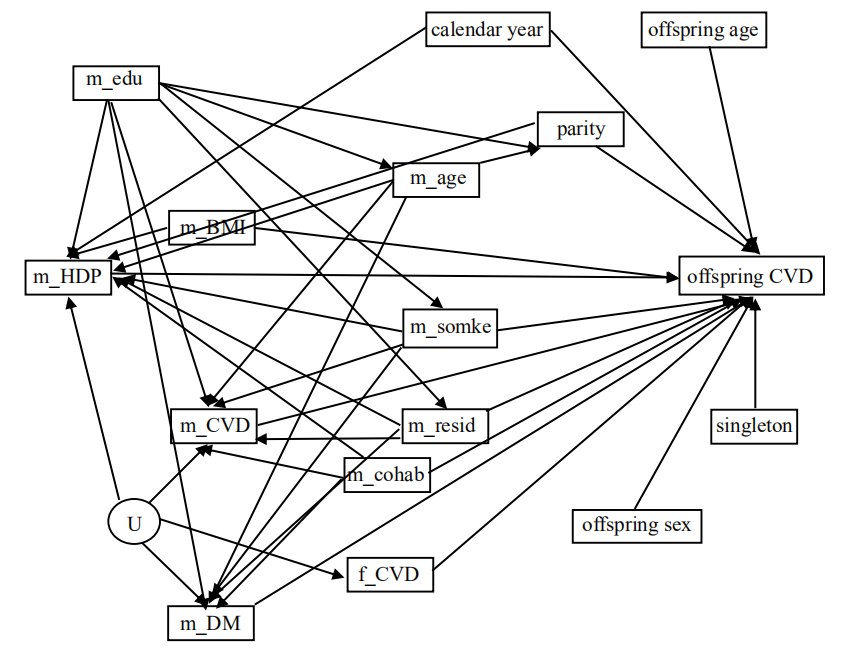
**

^a^ m_HPD: maternal hypertension disorders, m_age: maternal age, m_smoke: maternal smoking, m_edu: maternal education, m_cohab: maternal cohabitation, m_resid: maternal residence at birth, m_BMI: maternal BMI, m_CVD: maternal history of CVD before childbirth, m_DM: maternal history of diabetes before childbirth, f_CVD: paternal history of CVD before birth of the child, U: unmeasured variable
