# Supplementary material for: Maternal hypertensive disorder of pregnancy and offspring early-onset cardiovascular disease in childhood, adolescence, and young adulthood: A national population-based cohort study
Source: PLoS Med. 2021 Sep 28;18(9):e1003805. doi: 10.1371/journal.pmed.1003805 (PMC8478255; doi:10.1371/journal.pmed.1003805)
Supplement: S2 Fig — (DOCX) [file pmed.1003805.s012.docx]

**S2 Fig. Associations between maternal hypertensive disorders of pregnancy and early-onset CVD in offspring of sibling pairs**


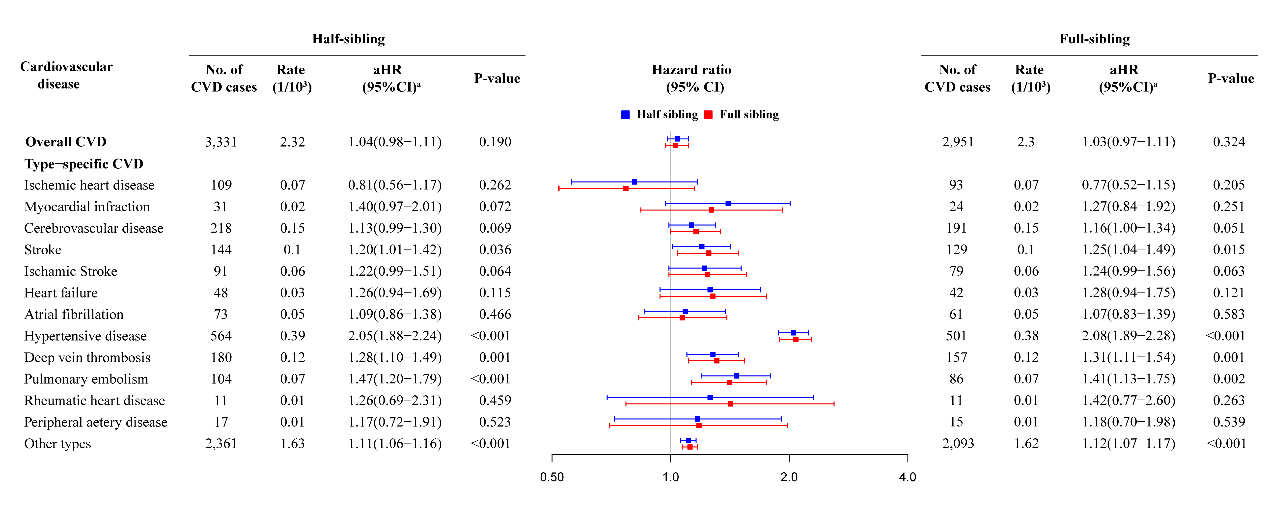


Abbreviations: HDP, hypertensive disorders of pregnancy; CVD, cardiovascular disease; cHR, crude hazard ratio; aHR, adjusted hazard ratio.

Half-sibling: sibling pairs of offspring born to same mother; full-sibling: sibling pairs of offspring born to same father and mother.

Adjusted for calendar year, sex, singleton status, parity, maternal age, maternal smoking, maternal education, maternal cohabitation, maternal income at birth, maternal residence at birth, maternal history of CVD and diabetes before childbirth, and paternal history of CVD before childbirth.
