# Supplementary material for: Maternal hypertensive disorder of pregnancy and offspring early-onset cardiovascular disease in childhood, adolescence, and young adulthood: A national population-based cohort study
Source: PLoS Med. 2021 Sep 28;18(9):e1003805. doi: 10.1371/journal.pmed.1003805 (PMC8478255; doi:10.1371/journal.pmed.1003805)
Supplement: S3 Fig — (DOCX) [file pmed.1003805.s013.docx]

**S3 Fig. Associations between maternal hypertensive disorder of pregnancy and early-onset CVD in offspring, according to the timing of the maternal HDP diagnosis**

**
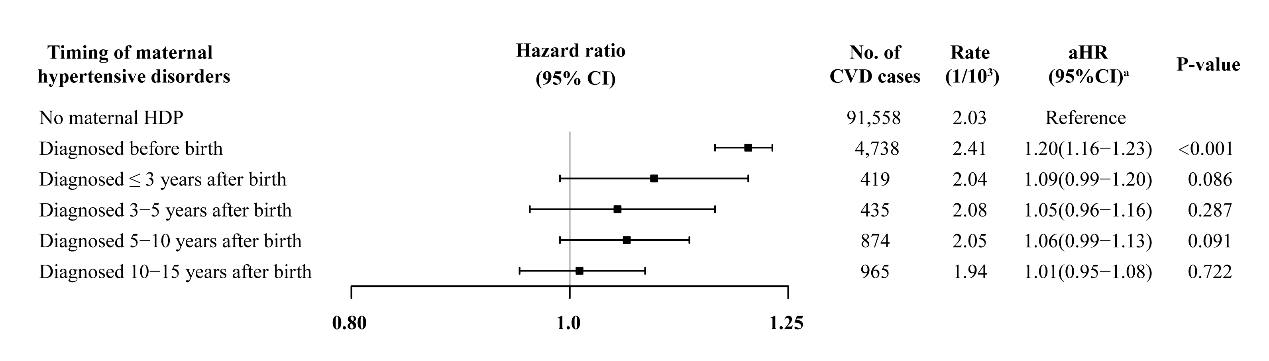
**

Abbreviations: HDP, hypertensive disorders of pregnancy; CVD, cardiovascular disease; cHR, crude hazard ratio; aHR, adjusted hazard ratio.

Adjusted for calendar year, sex, singleton status, parity, maternal age, maternal smoking, maternal education, maternal cohabitation, maternal country of origin, maternal income at birth, maternal BMI, maternal residence at birth, maternal history of CVD and diabetes before childbirth, and paternal history of CVD before childbirth.
