# Supplementary figures and images for: Maternal hypertensive disorder of pregnancy and offspring early-onset cardiovascular disease in childhood, adolescence, and young adulthood: A national population-based cohort study
Source: PLoS Med. 2021 Sep 28;18(9):e1003805. doi: 10.1371/journal.pmed.1003805 (PMC8478255; doi:10.1371/journal.pmed.1003805)

**S4 Fig. The log-minus-log survival curve**


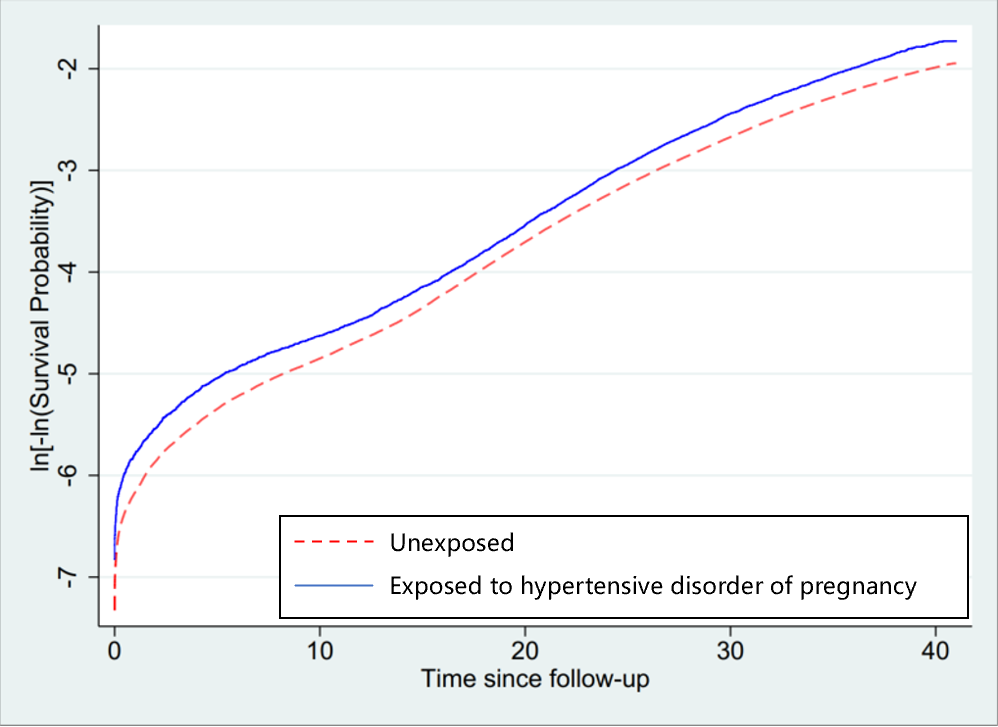

Supplement: S4 Fig — (DOCX) [file pmed.1003805.s014.docx]
