# Supplementary material for: Maternal hypertensive disorder of pregnancy and offspring early-onset cardiovascular disease in childhood, adolescence, and young adulthood: A national population-based cohort study
Source: PLoS Med. 2021 Sep 28;18(9):e1003805. doi: 10.1371/journal.pmed.1003805 (PMC8478255; doi:10.1371/journal.pmed.1003805)
Supplement: Attachment — Submitted filename: PMEDICINE-D-21-00753R1_rebuttal letter.docx [file pmed.1003805.s015.docx]

Richard Turner

Senior Editor, PLOS Medicine

**Manuscript ID: PMEDICINE-D-21-00753R1**

**MS TITLE: Maternal hypertensive disorder of pregnancy and offspring early-onset cardiovascular disease in childhood, adolescence, and young adulthood: A national population-based cohort study**

Dear Dr. Turner,

We thank the Editors and Reviewers for their careful review of our work and for the opportunity to revise our manuscript according to their suggestions.

In line with your instructions, we have provided a numbered point-by-point response to the comments provided by the Editors and Reviewers. We have tracked all changes in the revised manuscript.

We believe that the manuscript has improved considerably and hope you will find it suitable for publication in PLOS Medicine.

Sincerely,

Yongfu Yu

On behalf of all authors

Department of Biostatistics, School of Public Health, and The Key Laboratory of Public Health Safety of Ministry of Education, Fudan University, Shanghai, China/ Department of Clinical Medicine - Department of Clinical Epidemiology, Aarhus University, Aarhus, Denmark

Email: yoyu@clin.au.dk,

Tel: +45 71316566

**Suggestions from Editors**

**E1.** In the abstract, please quote summary demographic details for study participants.

**[Response]**

We have added some demographic details for study participants in the abstract.

**Abstract (Page 3, Line 59-61)**

“The median follow-up time was 18.37 years, and 51.3% of the participants were males. A total of 4532 offspring in the exposed group (2.47 per 1000 person-years) and 94 457 in the unexposed group (2.03 per 1000 person-years) were diagnosed with CVD.”

**E2.** Please add a new final sentence to the "Methods and findings" subsection of your abstract, which should begin "Study limitations include ..." or similar and quote 2-3 of the study's main limitations.

**[Response]**

We have acknowledged the main limitation of this study in the abstract:

**Abstract (Page 4, Line 72-74)**

“Study limitations include the lack of information on certain potential confounders (smoking, physical activity, alcohol consumption, etc.) and limited generalizability in other countries with varying disparities in healthcare.”

**E3.** Please add bullets to the individual points in your author summary.

**[Response]**

As suggested, we have added bullets to the individual points in ‘author summary’ part.

**E4.** Early in the Methods section, please state whether the study had a protocol or prespecified analysis plan, and if so attach the relevant document(s) as supplementary files, referred to in the text. Please highlight non-prespecified analyses.

**[Response]**

We have provided our protocol in the appendix and mentioned it in the Methods section:

**Method section (Page 8, Line 147-148)**

“The detailed study protocol is available in Appendix 2.”

**E5.** At line 186, would " ... preplanned sensitivity analyses:" be appropriate?

**[Response]**

We have rephrased it as “We performed the following sensitivity analyses:”.

**E6.** At line 330 and any other relevant points, please make that "... also worth mentioning.".

**[Response]**

Thanks for the suggestion, and we have revised as “also worth mentioning”.

**E7.** Throughout the text, please remove spaces from within your reference call-outs (e.g., "... undeveloped countries [3,4].").

**[Response]**

We have removed spaces between references throughout the text.

**E8.** At the end of the main text, please remove information on funding, competing interests and data availability. In the event of publication, this information will appear in the article metadata, via entries in the submission form.

**[Response]**

We have removed information on funding, competing interests and data availability from the main text.

**E9.** In the reference list, please remove the information on competing interests and so on from reference 4, and any other relevant references.

**[Response]**

Thanks, we have removed information on competing interests from reference 4 and all other references.

**E10.** Noting reference 26, please ensure that all references have full access information.

**[Response]**

Thanks for pointing it out. We have checked all references, and all references, including reference 26, have full access information now.

**E11.** Noting table 1, please substitute "sex" for "gender" where appropriate.

**[Response]**

Thanks for the suggestion, and gender has been replaced by ‘sex’ in table 1.

**E12.** Please move figure S1 to the main body of the paper.

**[Response]**

We have moved figure S1 to the main body of the paper as figure 1.

**E13.** Please add a completed checklist for the most appropriate reporting guideline, e.g., STROBE, labelled "S1_STROBE_Checklist" or similar and referred to as such in your Methods section. In the checklist, please refer to individual items by section (e.g., "Methods") and paragraph number rather than by line or page numbers, as the latter generally change in the event of publication.

**[Response]**

We have provided STROBE checklist in the appendix and added a relevant statement in the Methods section:

**Method section (Page 8, Line 148-150)**

“We have followed the Strengthening the Reporting of Observational Studies in Epidemiology (STROBE) guidelines (STROBE Checklist is provided in Appendix 3).”

**Comments from Reviewers**

**Reviewer 1**

**Comments:**

This is a thorough and comprehensive analysis, applying an appropriate and rigorous statistical methodology.

An extensive array of sensitivity and subgroup analyses have been completed demonstrating robustness and providing confidence in the results.

The authors have also adequately acknowledged the main study limitations within the discussion section.

**[Response]**

We appreciate the reviewer’s positive comments.

**Reviewer 2**

**Comments:**

This is a very interesting study involving a very large number of subjects exposed to a variety of hypertensive disorders of pregnancy including preeclampsia and eclampsia. The authors also divide preeclampsia in early onset and late onset. The authors conclude that exposure to preeclampsia is associated with a variety of adverse cardiovascular events over a broad range of ages;up to the age of 40 years. The authors also report that maternal history of diabetes and cardiovascular disease are risk factors for cardiovascular disease in the offspring. The data are robust, the analysis very detail and I suggest that the manuscript gets published in its current form.

**[Response]**

We appreciate the reviewer’s positive comments.

**Reviewer 3**

**General Comments:**

This is a good study and gives unique information from a large population registry in Denmark. There were only a few issues that concerned me and may require the authors to remove or explain.

**[Response]**

We appreciate the reviewer’s positive comments.

**R3.1.** The conclusion that treatment of the maternal hypertensive disorder of pregnancy may reduce the burden of CVD in offspring is unfounded and cannot be stated and should be removed.

**[Response]**

We agree with the reviewer and we have now removed the statement as suggested.

**R3.2.** How many in the cohort were lost to follow-up due to emigration and how would this confound the results due to lost data?

**[Response]**

Thanks for the comment. A total of 68,675 (2.76%) offspring were lost to follow-up due to emigration in our study, which was relatively small compared to our large sample size. We also observed the frequency of loss to follow-up due to emigration was quite close between the exposed group (2.79%) and the non-exposed group (2.02%). In our analyses, the participants who lost to follow-up due to emigration were censored at the date of emigration, which is a widely used approach to deal with emigration in the register-based study [1-9]. Therefore, it is less likely that the results would be changed significantly due to loss to follow-up. We have added information on loss to follow-up due to emigration in our manuscript as follows:

**Results section (Page 11, Line 215-217)**

“A total of 88 275 offspring (3.55%) were censored during the follow-up, of which 68 675 (2.76%) were due to emigration and 19 600 (0.79%) were due to non-cardiovascular death.”

**R3.3.** The CVD endpoints are very inclusive and include both arterial and venous disease. Although these may all be linked to disorders of coagulation, they have many other different risk factors during life and this may act as a confounder. How can this be managed differentially?

**[Response]**

Thank you for the comments. Given the exploratory nature of the study, we are aiming to provide a more detailed overview on the association of maternal HDP with a finer categorization of various type-specific CVDs, based on the grouping of ICD codes. We agreed that different subtypes of CVD would result from different pathophysiological mechanisms and have various life-time risk factors. In line with these facts, our findings showed that the magnitude of the associations between maternal HDP and type-specific CVDs differed very much across different CVD subtypes (different HR values, Table 2 in the main text). However, given that we do not have information on many other different risk factors of arterial and venous disease, we were not able to include them as potential confounders, and therefore, to manage their effects differentially is beyond the scope of our study. However, our observations may still provide useful information calling for further research investigating the potential mechanisms for arterial and venous disease, taking into consideration prenatal exposure to maternal HDP. We are certainly willing to exclude arterial and venous disease as CVDs, if the reviewer thinks it would be more appropriate. We have added the following information to the discussion section:

**Discussion section (Page 15, Line 297-301)**

“The differences in the association between maternal HDP and type-specific CVDs in offspring may be due to complex pathophysiology and the effects of various future risk factors for the development of type-specific CVDs [37]. Further investigation on the underlying mechanisms and to explore the effects of other different risk factors during life for specific CVDs are warranted.”

**R3.4.** Rheumatic heart disease was included as a CVD. This disorder is an infective disease (streptococcus) with a genetic risk factor component very different to the other CVD studied. I note there were only a few patients in this group, but I wonder why this group was included?

**[Response]**

We appreciate this insightful comment. We totally agree with the reviewer that the etiology of rheumatic heart disease might be very different from that of other CVDs. Again, there were only a few patients, and the analysis of this group separately may not be very informative. As expected, when we performed an additional analysis that excluded rheumatic heart disease from CVD outcomes, we obtained similar results (adjusted HR, 1.23; 95% CI, 1.19 to 1.26) as those from the main analyses in the manuscript. Given the exploratory nature of the study, we are aiming to provide a more detailed overview on the association of maternal HDP with a finer categorization of various type-specific CVDs, based on the grouping of ICD codes. Therefore, at this stage, we would like to keep this group in the Table. We are certainly willing to exclude rheumatic heart disease as a CVD, if the reviewer thinks it would be more appropriate.

**R3.5.** In the discussion, inclusion of smoking as a potentially lifestyle risk factor in the cohort should be mentioned alongside physical activity and alcohol use as smoking is one of the "big five" CVD risk factors.

**[Response]**

Thanks for the comment. Unfortunately, we have no available information on offspring smoking status in registries. We have acknowledged this in the limitation paragraph as suggested:

**Limitation paragraph in Discussion section (Page 17, Line 346):**

“First, we could not exclude the possibility of residual confounding due to lack of information on certain important confounders, such as smoking status, physical activity, alcohol use, diet, and other lifestyle factors [2,44].”

**Reference**

1. Yu Y, Arah OA, Liew Z, Cnattingius S, Olsen J, Sorensen HT, et al. Maternal diabetes during pregnancy and early onset of cardiovascular disease in offspring: population based cohort study with 40 years of follow-up. BMJ. 2019;367:l6398. Epub 2019/12/06. doi: 10.1136/bmj.l6398. PubMed PMID: 31801789; PubMed Central PMCID: PMCPMC6891797.
2. Yu Y, Liew Z, Cnattingius S, Olsen J, Vestergaard M, Fu B, et al. Association of Mortality With the Death of a Sibling in Childhood. JAMA Pediatr. 2017;171(6):538-45. Epub 2017/04/25. doi: 10.1001/jamapediatrics.2017.0197. PubMed PMID: 28437534; PubMed Central PMCID: PMCPMC5540009.
3. Crump C, Sundquist J, Sundquist K. Association of preterm birth with lipid disorders in early adulthood: A Swedish cohort study. PLOS Medicine. 2019;16(10):e1002947. Epub 2019/10/19. doi: 10.1371/journal.pmed.1002947. PubMed PMID: 31626652; PubMed Central PMCID: PMCPMC6799885.
4. Iversen L, Fielding S, Lidegaard O, Morch LS, Skovlund CW, Hannaford PC. Association between contemporary hormonal contraception and ovarian cancer in women of reproductive age in Denmark: prospective, nationwide cohort study. BMJ. 2018;362:k3609. Epub 2018/09/28. doi: 10.1136/bmj.k3609. PubMed PMID: 30257920; PubMed Central PMCID: PMCPMC6283376.
5. Dalsgaard S, Ostergaard SD, Leckman JF, Mortensen PB, Pedersen MG. Mortality in children, adolescents, and adults with attention deficit hyperactivity disorder: a nationwide cohort study. Lancet. 2015;385(9983):2190-6. Epub 2015/03/03. doi: 10.1016/S0140-6736(14)61684-6. PubMed PMID: 25726514.
6. Sun Y, Christensen J, Hviid A, Li J, Vedsted P, Olsen J, et al. Risk of febrile seizures and epilepsy after vaccination with diphtheria, tetanus, acellular pertussis, inactivated poliovirus, and Haemophilus influenzae type B. JAMA. 2012;307(8):823-31. Epub 2012/02/24. doi: 10.1001/jama.2012.165. PubMed PMID: 22357833.
7. Sujan AC, Rickert ME, Oberg AS, Quinn PD, Hernandez-Diaz S, Almqvist C, et al. Associations of Maternal Antidepressant Use During the First Trimester of Pregnancy With Preterm Birth, Small for Gestational Age, Autism Spectrum Disorder, and Attention-Deficit/Hyperactivity Disorder in Offspring. JAMA. 2017;317(15):1553-62. Epub 2017/04/19. doi: 10.1001/jama.2017.3413. PubMed PMID: 28418479; PubMed Central PMCID: PMCPMC5875187.
8. Li J, Laursen TM, Precht DH, Olsen J, Mortensen PB. Hospitalization for mental illness among parents after the death of a child. N Engl J Med. 2005;352(12):1190-6. Epub 2005/03/25. doi: 10.1056/NEJMoa033160. PubMed PMID: 15788495.
9. Razaz N, Villamor E, Muraca GM, Bonamy AE, Cnattingius S. Maternal obesity and risk of cardiovascular diseases in offspring: a population-based cohort and sibling-controlled study. Lancet Diabetes Endocrinol. 2020;8(7):572-81. Epub 2020/06/20. doi: 10.1016/S2213-8587(20)30151-0. PubMed PMID: 32559473.
