# Supplementary material for: Maternal hypertensive disorder of pregnancy and offspring early-onset cardiovascular disease in childhood, adolescence, and young adulthood: A national population-based cohort study
Source: PLoS Med. 2021 Sep 28;18(9):e1003805. doi: 10.1371/journal.pmed.1003805 (PMC8478255; doi:10.1371/journal.pmed.1003805)
Supplement: Attachment — Submitted filename: PMEDICINE-D-21-00753R2_rebuttal letter.docx [file pmed.1003805.s016.docx]

Richard Turner

Senior Editor, PLOS Medicine

**Manuscript ID: PMEDICINE-D-21-00753R2**

**MS TITLE: Maternal hypertensive disorder of pregnancy and offspring early-onset cardiovascular disease in childhood, adolescence, and young adulthood: A national population-based cohort study**

Dear Dr. Turner,

We thank the Editors and Reviewers for their careful review of our work and for the opportunity to revise our manuscript according to their suggestions.

In line with your instructions, we provide below a numbered point-by-point response to each comment provided by the Editors and Reviewers. We have tracked all changes in the revised manuscript.

We believe that the manuscript has improved considerably and hope you will find it suitable for publication in PLOS Medicine.

Sincerely,

Yongfu Yu

On behalf of all authors

Department of Biostatistics, School of Public Health, and The Key Laboratory of Public Health Safety of Ministry of Education, Fudan University, Shanghai, China/ Department of Clinical Medicine - Department of Clinical Epidemiology, Aarhus University, Aarhus, Denmark

Email: yu@fudan.edu.cn, yoyu@clin.au.dk

Tel: +45 71316566

**Requests from Editors:**

**E1.** In the abstract and throughout the results section, please quote p values alongside 95% CI, where available.

**[Response]**

We have added P values alongside 95% CI throughout the manuscript, including the abstract and results section, all tables and figures.

**E2.** At line 62, please make that "... an increased risk of early onset CVD ..." (the "23%" is implicit in the quoted HR).

**[Response]**

We have revised this sentence at line 62 and removed “23%” as suggested.

**Abstract (Page 4, Line 61-63)**

“We found that exposure to maternal HDP was associated with an increased risk of early-onset CVD (hazard ratio [HR]: 1.23; 95% CI = 1.19 to 1.26; P<0.001).”

**E3.** At line 73, please make that "... including smoking ..." and remove "etc.".

**[Response]**

We have revised this sentence at line 73 as suggested.

**Abstract (Page 4, Line 73-75)**

“Study limitations include the lack of information on certain potential confounders (including smoking, physical activity, and alcohol consumption) and limited generalizability in other countries with varying disparities in healthcare.”

**E4.** At line 88, please make that "extent".

**[Response]**

Thanks so much, we have corrected this word as “extent” at line 88.

**E5.** At line 95, we suggest making that "(67% increased risk)" for clarity.

**[Response]**

We have revised it as suggested to make it clear.

**Author Summary (Page 5, Line 96-98)**

“We found that individuals born to mothers with HDP had a 23% increased risk of early-onset CVD in offspring, especially of those mothers with a history of CVD (67% increased risk) or diabetes (56% increased risk).”

**E6.** Thank you for including the study protocol. Were there any non-prespecified analyses?

**[Response]**

All analyses were pre-planned by the authors at the time of conception and design of the study, and we have revised our description in the manuscript:

**Methods (Page 7, Line 144-145)**

“The detailed prespecified study protocol is available in S1_study_protocol.”

**E7.** At lines 230 and 232, the upper bound of the 95% CI is quoted as "1.26" and "1.27". Please check that both numbers are correct.

**[Response]**

We have checked data carefully, and both “1.26” and “1.27” were correct.

In line 233 (line 230 in the previous version): “We found offspring of mothers with both HDP and history of CVD had a higher risk of early-onset CVD (HR, 1.67; 95% CI, 1.41 to 1.98), compared to offspring born to mothers with HDP alone (HR, 1.23; 95% CI, 1.19 to 1.26).” This analysis was to examine joint effect of HDP and a history of CVD on offspring CVD. A hazard ratio of 1.23 was calculated in offspring whose mothers didn’t have a history of CVD.

In line 236 (line 232 in the previous version): ”Offspring born to mothers with HDP and history of diabetes also tended to have a higher risk of early-onset CVD (HR, 1.56; 95% CI, 1.34 to 1.83), compared to offspring of mothers with HDP alone (HR, 1.23; 95% CI, 1.19 to 1.27) (Table 3).” The analysis was to examine joint effect of HDP and a history of diabetes on offspring CVD. A hazard ratio of 1.23 was calculated in offspring whose mothers didn’t have a history of diabetes.

The two analyses have different objectives. The HRs from the two analyses are coincidentally the same, while the upper bounds of CI are slightly different (1.26 vs. 1.27).

**E8.** Please revisit the author list for reference 28 so that the format matches that of the other references.

**[Response]**

Thank you very much, we have revised the author list in reference 28.

**E9.** We suggest breaking the study protocol and STROBE checklist out into separate attached files, labelled and referred to in the text as "S2_STROBE_Checklist" and similar.

**[Response]**

We have break the study protocol and STROBE checklist out into separate attached files and renamed as S1_study_Protocol and S2_STROBE_Checklist.

**Comments from Reviewers**

**Reviewer 4**

**Comments:**

This study set out to elucidate the association between maternal hypertensive diseases of pregnancy and early-onset CVD in childhood and youth (before 40 years of age). It was conducted as a population-based cohort study of 2.5 million individuals. The study found that maternal HDP (defined as preeclampsia, eclampsia, gestational hypertension or pre-existing hypertension) increased the risk of early CVD by 23%. Specific risk increases were found for MI and hypertension. The association was strongest for early-onset and severe preeclampsia with a 48% risk increase for CVD.

The study however lacked information on some potential confounders such smoking and physical activity, yet a range of other covariables were available.

**Major Comments:**

This study is unique and has beautiful statistical strength in having such a vast register-based material with follow up over several decades. I congratulate the authors on this feat. Further extensive subgroup analyses have been performed including sibship analyses, timing of delivery and stratified analyses by offspring sex, singleton/gemelli, maternal parity and a range of maternal covariables.

This is a rather extraordinary study which warrants immediate publication and follow-up studies.

**[Response]**

We appreciate the Reviewer’s positive comments.

**Minor comments:**

Perhaps I lack understanding of the nature of sibship analysis, but I believe maybe some wording is missing from lines 242-247, to fully understand the implications of the analyses?

**[Response]**

Thanks so much for the comment. As suggested, we have added more description of sibship analyses in line 243-245 in result section to clarify this as follows:

**Results section (Page 12, Line 245-247)**

“Sibship analyses restricting offspring to sibling pairs with same mother but different father (half-sibling) or sibling pairs with same mother and father (full-sibling) showed the increased risks of most type-specific CVDs (Figure S2), …”
